# Supplementary material for: Circulating Factors as Potential Biomarkers of Cardiovascular Damage Progression Associated with Type 2 Diabetes
Source: Proteomes. 2024 Oct 11;12(4):29. doi: 10.3390/proteomes12040029 (PMC11503308; doi:10.3390/proteomes12040029)

Supplementary material

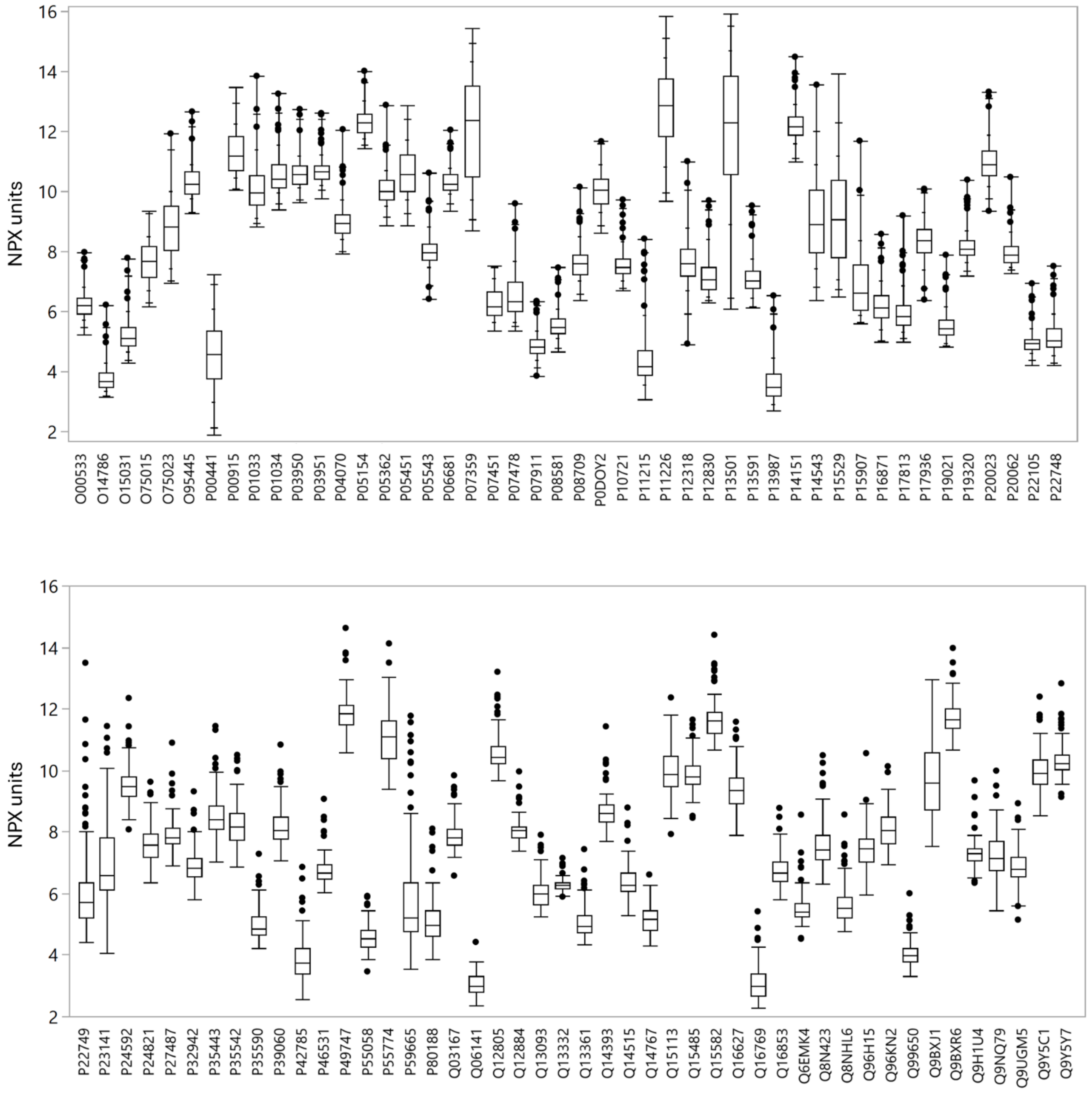

Figure S1: Box plot with the distribution of Normalized Protein eXpression (NPX) in the overall samples. Data are expressed as Olink's arbitrary unit in Log_2 scale. Please see **Table S3** for values in each group.

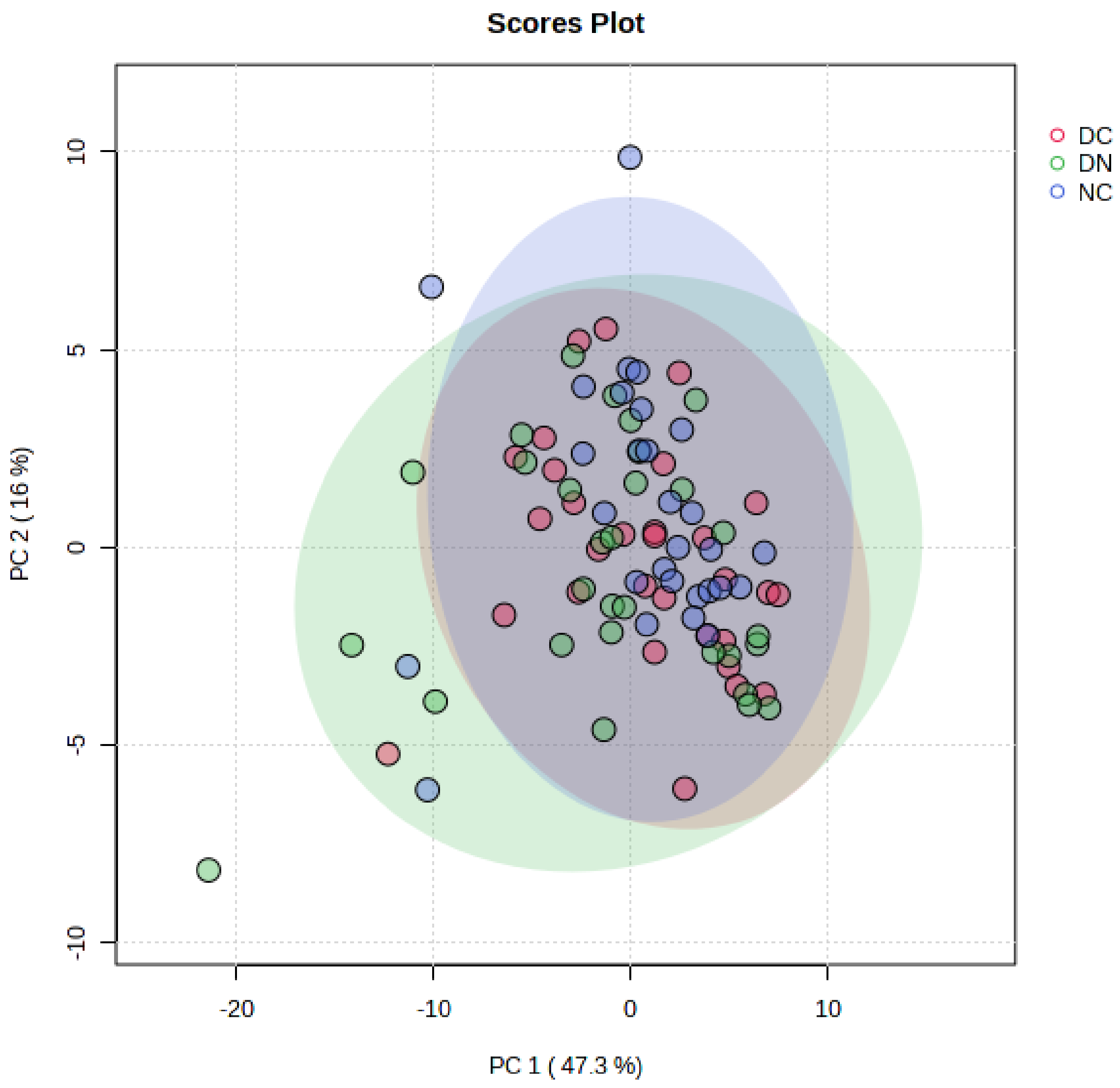

Figure S2: PCA analysis using the 92 measured proteins, indicating no specific distinction between the three patients' groups.

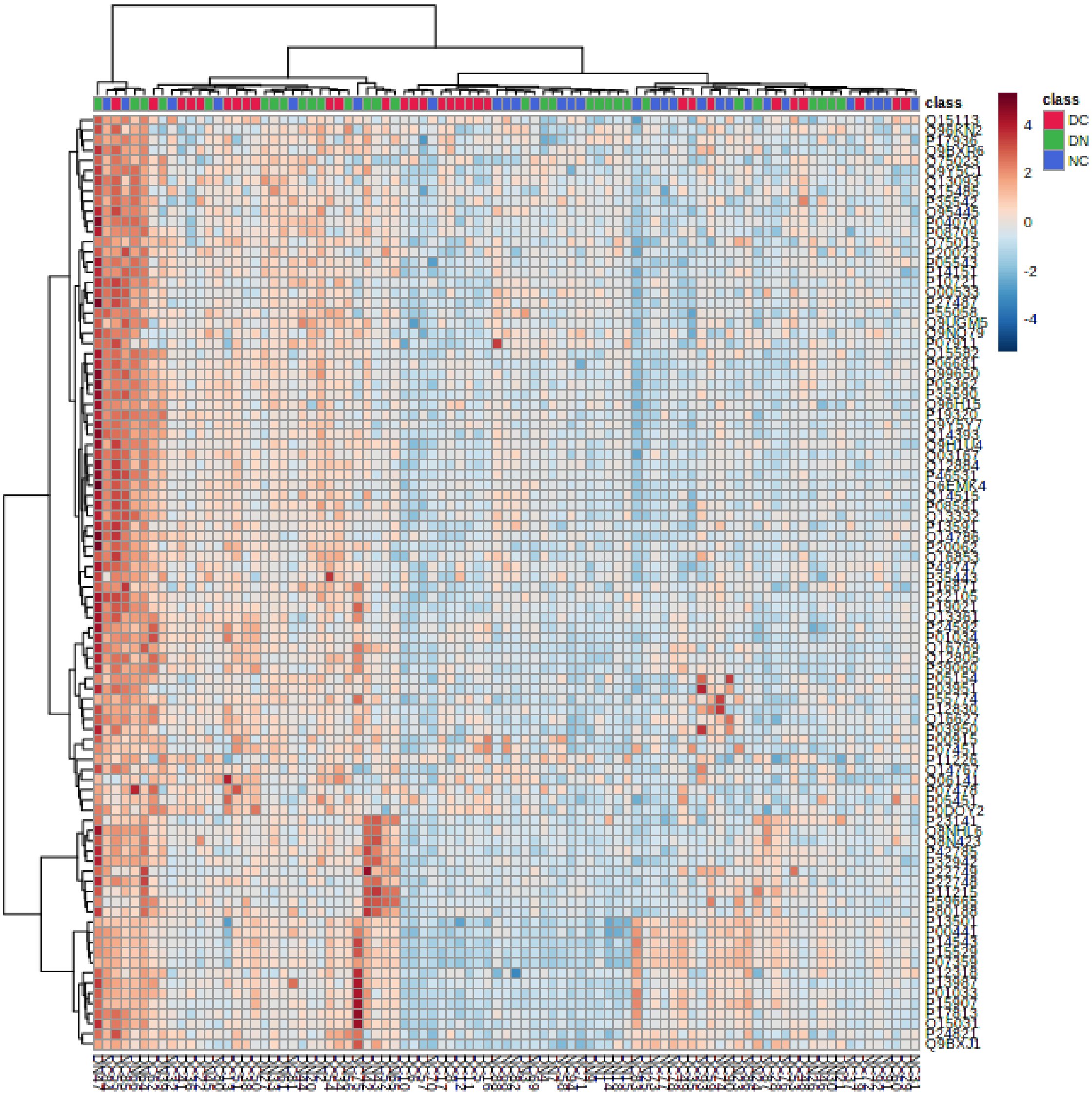

Figure S3: Heatmap from cluster analysis using the 92 measured proteins, indicating no specific distinction between the three patients' groups.

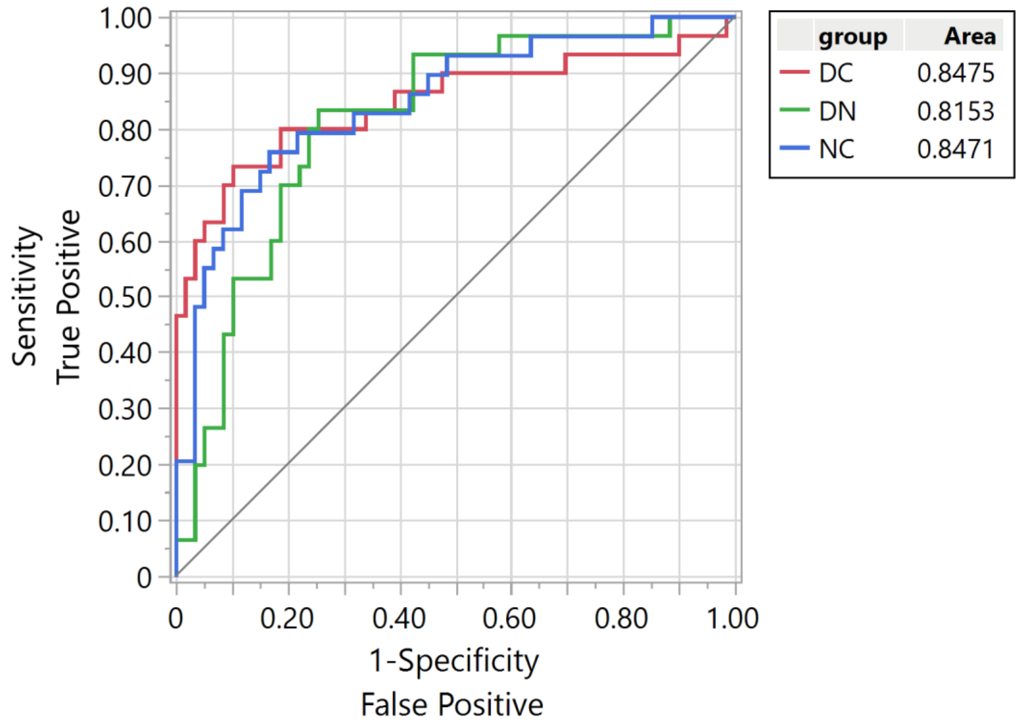

Whole Model Test

Model	-LogLikelihood	DF	ChiSquare	Prob>ChiSq
Difference	26.438320	12	52.87664	<.0001*
Full	71.326895			
Reduced	97.765215			

Source	Logworth	*P* Value
P05451	1.857	0.01389
P23141	1.340	0.04568
P0DOY2	1.224	0.05964
P32942	1.044	0.09040
P06681	0.529	0.29612
P42785	0.056	0.87955

Figure S4: ROC analysis obtained with nominal logistic plot using the six significant proteins for distinguishing among the three groups. The table below the graph indicates the effect summary with logworth and *p* value for each protein contribution.

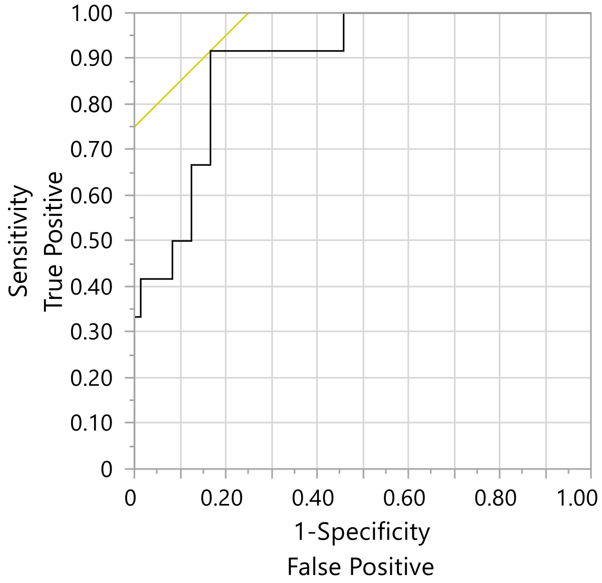

Nominal Logistic Fit for eGFR<60 ml/min - Effect Summary. AUC= 0.89 (whole model test: *p*=0.0003)

Source	Logworth	*P* Value
P0DOY2 (IGLC2)	3.286	0.00052
P42785 (PRCP)	1.337	0.04598
P05451 (REG1A)	0.366	0.43077
P23141 (CES1)	0.175	0.66758
P32942 (ICAM3)	0.096	0.80158
P06681 (C2)	0.001	0.99704

Figure S5: ROC analysis obtained with nominal logistic plot using the six significant proteins on prediction of eGFR impairment (eGFR<60 ml/min), considering all the subjects together. The table below the graph indicates the effect summary with logworth and *p* value for each protein contribution.

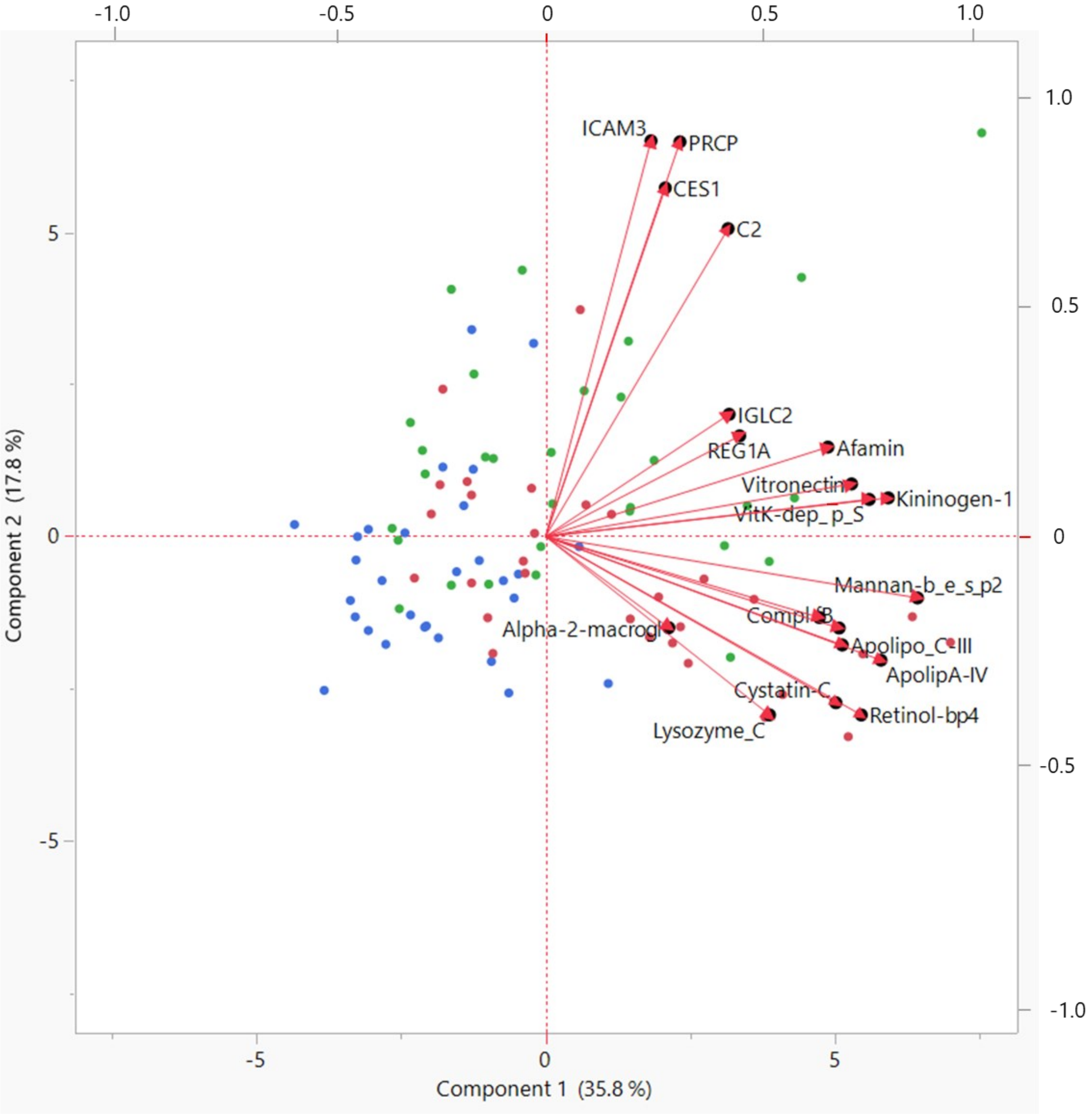

Figure S6. Biplot from PCA analysis on the 6 identified significant proteins together the 11 identified proteins found in a previous investigation on the same subjects using Multiplexed MRM-based proteomics [9]. The left and bottom axes show principal component scores; the top and right axes indicate the loadings. Red dots: DC group; green: DN group; blue: NC group.

Table S1. List of proteins analyzed using proximity extension assay (PEA).

Protein	Acronym	Uniprot ID
Angiogenin	ANG	P03950
Angiopoietin-related protein 3	ANGPTL3	Q9Y5C1
Alipoprotein M	APOM	O95445
Beta-Ala-His dipeptidase	CNDP1	Q96KN2
Beta-galactoside alpha-2,6-sialyltransferase 1	ST6GAL1	P15907
Cadherin-1	CDH1	P12830
Carbonic anhydrase 1	CA1	P00915
Carbonic anhydrase 3	CA3	P07451
Carbonic anhydrase 4	CA4	P22748
Cartilage acidic protein 1	CRTAC1	Q9NQ79
Cartilage oligomeric matrix protein	COMP	P49747
C-C motif chemokine 5	CCL5	P13501
C-C motif chemokine 14	CCL14	Q16627
C-C motif chemokine 18	CCL18	P55774
CD59 glycoprotein	CD59	P13987
Coagulation factor VII	F7	P08709
Coagulation factor XI	F11	P03951
Collagen alpha-1 (XVIII) chain	COL18A1	P39060
Complement C1q tumor necrosis factor-related protein 1	C1QTNF1	Q9BXJ1
Complement C2	C2	P06681
Complement factor H-related protein 5	CFHR5	Q9BXR6
Complement receptor type 2	CR2	P20023
Cystatin-C	CST3	P01034
Dipeptidyl peptidase 4	DPP4	P27487
EGF-containing fibulin-like extracellular matrix protein 1	EFEMP1	Q12805
Endoglin	ENG	P17813
Fetuin-B	FETUB	Q9UGM5
Ficolin-2	FCN2	Q15485
Glutaminyl-peptide cyclotransferase	QPCT	Q16769
Granulysin	GNLY	P22749
Growth arrest-specific protein 6	GAS6	Q14393
Hepatocyte growth factor receptor	MET	P08581
Ig lambda-2 chain C regions	IGLC2	P0CG05
Insulin-like growth factor-binding protein 3	IGFBP3	P17936

Protein	Acronym	Uniprot ID
Insulin-like growth factor-binding protein 6	IGFBP6	P24592
Integrin alpha-M	ITGAM	P11215
Intercellular adhesion molecule 1	ICAM1	P05362
Intercellular adhesion molecule 3	ICAM3	P32942
Interleukin-7 receptor subunit alpha	IL7R	P16871
Latent-transforming growth factor beta-binding protein 2	LTBP2	Q14767
Leukocyte immunoglobulin-like receptor subfamily B member 1	LILRB1	Q8NHL6
Leukocyte immunoglobulin-like receptor subfamily B member 2	LILRB2	Q8N423
Leukocyte immunoglobulin-like receptor subfamily B member 5	LILRB5	O75023
Lithostatine-1-alpha	REG1A	P05451
Liver carboxylesterase 1	CES1	P23141
Low affinity immunoglobulin gamma Fc region receptor II-a	FCGR2A	P12318
Low affinity immunoglobulin gamma Fc region receptor III-B	FCGR3B	O75015
L-selectin	SELL	P14151
Lymphatic vessel endothelial hyaluronic acid receptor 1	LYVE1	Q9Y5Y7
Lysosomal Pro-X carboxypeptidase	PRCP	P42785
Mannose-binding protein C	MBL2	P11226
Mast/stem cell growth factor receptor Kit	KIT	P10721
Membrane cofactor protein	CD46	P15529
Membrane primary amine oxidase	AOC3	Q16853
Metalloproteinase inhibitor 1	TIMP1	P01033
Microfibrillar-associated protein 5	MFAP5	Q13361
Multiple epidermal growth factor-like domains protein 9	MEGF9	Q9H1U4
Neural cell adhesion molecule 1	NCAM1	P13591
Neural cell adhesion molecule L1-like protein	CHL1	O00533
Neurogenic locus notch homolog protein 1	NOTCH1	P46531
Neuropilin-1	NRP1	O14786
Neutrophil defensin 1	DEFA1	P59665
Neutrophil gelatinase-associated lipocalin	LCN2	P80188
Nidogen-1	NID1	P14543
Oncostatin-M-specific receptor subunit beta	OSMR	Q99650
Peptidyl-glycine alpha-amidating monooxygenase	PAM	P19021
Phospholipid transfer protein	PLTP	P55058
Plasma serine protease inhibitor	SERPINA5	P05154
Platelet glycoprotein Ib alpha chain	GP1BA	P07359

Protein	Acronym	Uniprot ID
Platelet-activating factor acetylhidrolase	PLA2G7	Q13093
Plexin-B2	PLXNB2	O15031
Procollagen C-endopeptidase enhancer 1	PCOLCE	Q15113
Prolyl endopeptidase FAP	FAP	Q12884
Receptor-type tyrosine-protein phosphatase S	PTPRS	Q13332
Regenerating islet-derived protein 3-alpha	REG3A	Q06141
Serum amyloid A-4 protein	SAA4	P35542
SPARC-like protein 1	SPARCL1	Q14515
Superoxide dismutase [Cu-Zn]	SOD1	P00441
T-cell immunoglobulin and mucin domain-containing protein 4	TIMD4	Q96H15
Tenascin	TNC	P24821
Tenascin-X	TNXB	P22105
Thrombospondin-4	THBS4	P35443
Thyroxine-binding globulin	SERPINA7	P05543
Transcobalamin-2	TCN2	P20062
Transforming growth factor beta receptor type 3	TGFBR3	Q03167
Transforming growth factor-beta-induced protein ig-h3	TGFBI	Q15582
Trypsin-2	PRSS2	P07478
Tyrosine-protein kinase receptor Tie-1	TIEI1	P35590
Uromodulin	UMOD	P07911
Vascular cell adhesion protein 1	VCAM1	P19320
Vasorin	VASN	Q6EMK4
Vitamin K-dependent protein C	PROC	P04070

Table S2. Clinical and metabolic parameters of subjects in study.

	**DC (n=30)**	**DN (n=30)**	**NC (n=29)**
Gender (M/F)	27/3	22/8	21/8
Age (y)	65.8±8.7**	60.3±5.8†	65.5±6.1
Diabetes duration (y)	10.9±7.6***	1.9±0.9	---
BMI (kg/m^2)	29.5±3.9	29.4±3.3†	26.7±5.2‡
FPG (mg/dl)	143.2±43.0	144.9±36.7†††	94.1±12.7‡‡‡
HbA1c (%)	7.1±0.8	6.8±0.8†††	5.8±0.2‡‡‡
Total cholesterol (mg/dl)	152±36***	191±36††	155±41
LDL cholesterol (mg/dl)	82±32**	112±40††	84±31
HDL cholesterol (mg/dl)	42±10	48±9	53±16‡‡‡
Triglycerides (mg/dl)	167±80	139±73††	87±32‡‡‡

Data are expressed as mean ±SD.
For each continuous variable, symbols indicate significant differences between means by the Tukey's HSD test. ***p<0.001, **p<0.01, DC *vs* DN; †††p<0.001, ††p<0.01, †p<0.05, DN *vs* NC; ‡‡‡ p<0.001, ‡‡ p<0.01, ‡p<0.05, DC *vs* NC.

Abbreviations: BMI, body mass index; FPG, fasting plasma glucose; HbA1c, glycated hemoglobin; HDL, high density lipoprotein; LDL, low density lipoprotein.

Full information regarding patients' cohort has been previously published in Piarulli et al. (2022). Methods used for metabolic parameters determination are described in the mentioned paper. [Piarulli F, Banfi C, Brioschi M, Altomare A, Ragazzi E, Cosma C, Sartore G, Lapolla A. The Burden of Impaired Serum Albumin Antioxidant Properties and Glyco-Oxidation in Coronary Heart Disease Patients with and without Type 2 Diabetes Mellitus. *Antioxidants (Basel),* 2022;11(8):1501. doi: 10.3390/antiox11081501].

Table S3: Values of Normalized Protein eXpression (NPX) in the three groups of patients. Data are expressed as Olink's arbitrary unit in Log_2 scale.

Protein	NPX units (mean+ SD)		
	DC group	DN group	NC group
O00533	6.09±0.44	6.32±0.49	6.27±0.5
O14786	3.8±0.47	3.82±0.57	3.75±0.43
O15031	5.14±0.5	5.31±0.57	5.19±0.67
O75015	7.46±0.73	7.87±0.7	7.63±0.63
O75023	8.82±0.97	9.06±1.12	8.57±0.99
O95445	10.23±0.53	10.56±0.67	10.22±0.54
P00441	4.29±1.02	4.45±1.21	4.88±1.08
P00915	11.3±0.78	11.38±0.79	11.11±0.63
P01033	10.06±0.77	10.12±0.91	10.15±1.03
P01034	10.81±0.64	10.56±0.76	10.36±0.56
P03950	10.71±0.42	10.56±0.59	10.56±0.6
P03951	10.64±0.36	10.77±0.5	10.72±0.59
P04070	8.92±0.49	9.24±0.8	8.92±0.57
P05154	12.27±0.34	12.42±0.47	12.24±0.61
P05362	10.07±0.46	10.22±0.72	9.91±0.53
P05451	11.08±0.76	10.66±0.74	10.17±0.61
P05543	7.93±0.49	8.27±0.69	7.87±0.61
P06681	10.39±0.32	10.49±0.48	10.1±0.42
P07359	11.8±1.79	12.16±1.78	12.38±1.66
P07451	6.35±0.59	6.25±0.56	6.25±0.51
P07478	6.89±0.86	6.53±0.82	6.24±0.54
P07911	4.67±0.44	4.98±0.33	4.93±0.45
P08581	5.46±0.48	5.66±0.48	5.54±0.41
P08709	7.6±0.48	7.91±0.73	7.41±0.55
P0DOY2	10.37±0.63	10.03±0.57	9.76±0.58
P10721	7.41±0.55	7.75±0.58	7.61±0.55
P11215	4.31±0.91	4.86±1.56	4.25±0.49
P11226	12.6±1.28	13.03±1.41	12.78±1.23
P12318	7.64±0.63	7.79±0.84	7.64±1.13
P12830	7.32±0.66	7.21±0.67	7.08±0.78
P13501	11.59±2.5	12.06±2.52	12.52±1.63
P13591	7.06±0.62	7.11±0.63	7.18±0.61
P13987	3.56±0.57	3.58±0.74	3.65±0.82
P14151	12.01±0.46	12.47±0.62	12.2±0.6
P14543	8.83±1.3	8.98±1.28	9.15±1.47
P15529	8.78±1.44	9.11±1.59	9.39±1.65
P15907	6.77±0.91	7±1.05	7.07±1.32
P16871	6.06±0.65	6.28±0.63	6.24±0.72
P17813	5.83±0.6	6.02±0.62	6.11±0.85
P17936	8.15±0.67	8.6±0.76	8.28±0.62
P19021	5.48±0.5	5.55±0.64	5.57±0.55
P19320	8.2±0.5	8.28±0.7	8.1±0.52
P20023	10.87±0.62	11.23±0.73	10.84±0.73

Protein	NPX units (mean+ SD)		
	DC group	DN group	NC group
P20062	8±0.46	8.02±0.6	7.91±0.43
P22105	4.86±0.41	5.09±0.49	5.04±0.44
P22748	5.1±0.54	5.36±0.8	5.02±0.39
P22749	6.19±1.33	6.52±2.17	5.97±1.18
P23141	6.88±1.03	7.93±1.65	6.36±1.23
P24592	9.73±0.61	9.47±0.78	9.45±0.49
P24821	7.66±0.63	7.52±0.66	7.6±0.62
P27487	7.73±0.51	8.17±0.66	7.91±0.56
P32942	6.84±0.47	7.24±0.72	6.72±0.45
P35443	8.52±0.92	8.69±0.85	8.44±0.57
P35542	8.24±0.82	8.46±0.71	8.04±0.74
P35590	4.94±0.4	5.14±0.58	4.9±0.5
P39060	8.39±0.66	8.19±0.73	8.02±0.58
P42785	3.81±0.53	4.29±0.93	3.57±0.63
P46531	6.74±0.44	6.91±0.58	6.71±0.44
P49747	11.78±0.68	11.91±0.71	11.95±0.62
P55058	4.5±0.47	4.67±0.47	4.58±0.44
P55774	11.19±0.79	11.2±0.75	10.97±1.11
P59665	5.98±1.81	6.44±2.43	5.16±0.68
P80188	5.17±0.62	5.37±1.12	4.86±0.52
Q03167	7.88±0.5	7.96±0.57	7.86±0.55
Q06141	3.1±0.43	3.02±0.3	2.96±0.31
Q12805	10.74±0.65	10.55±0.7	10.55±0.63
Q12884	8.01±0.42	8.17±0.46	8.03±0.32
Q13093	5.96±0.52	6.27±0.57	5.92±0.43
Q13332	6.26±0.2	6.31±0.25	6.27±0.2
Q13361	5.11±0.54	5.12±0.63	5.03±0.47
Q14393	8.61±0.49	8.8±0.7	8.69±0.55
Q14515	6.3±0.53	6.56±0.63	6.35±0.55
Q14767	5.15±0.44	5.18±0.45	5.15±0.44
Q15113	10.1±0.68	9.72±0.87	10.1±0.76
Q15485	9.77±0.52	9.99±0.65	9.81±0.54
Q15582	11.63±0.57	11.97±0.73	11.49±0.55
Q16627	9.45±0.68	9.39±0.69	9.38±0.83
Q16769	3.16±0.48	3.25±0.66	2.95±0.57
Q16853	6.87±0.58	6.82±0.57	6.68±0.54
Q6EMK4	5.52±0.49	5.65±0.71	5.44±0.48
Q8N423	7.68±0.8	7.94±1.11	7.43±0.67
Q8NHL6	5.58±0.49	5.89±0.86	5.54±0.57
Q96H15	7.42±0.6	7.64±0.89	7.46±0.55
Q96KN2	8.01±0.6	8.36±0.69	7.88±0.49
Q99650	4.02±0.32	4.19±0.48	3.95±0.39
Q9BXJ1	9.68±1	9.8±0.97	9.54±1.28
Q9BXR6	11.73±0.6	11.83±0.68	11.71±0.53
Q9H1U4	7.25±0.5	7.52±0.59	7.22±0.41
Q9NQ79	6.91±0.72	7.38±0.82	7.52±0.77

Protein	NPX units (mean+ SD)		
	DC group	DN group	NC group
Q9UGM5	6.7±0.63	7.11±0.74	6.82±0.59
Q9Y5C1	9.83±0.6	10.24±0.76	9.89±0.63
Q9Y5Y7	10.33±0.5	10.41±0.66	10.23±0.52

Table S4: Linear correlation between the six identified significant proteins with the *p* values for statistical significance of the correlations. See also Figure 3 in the manuscript. *P* values highlighted in red color and marked with an asterisk indicate significant correlations.

Variable	by Variable	Correlation *r*	Count	Lower 95%	Upper 95%	*P*
P06681 (C2)	P05451 (REG1A)	0.5403	89	0.3741	0.6728	<.0001*
P0DOY2 (IGLC2)	P05451 (REG1A)	0.5456	89	0.3806	0.6770	<.0001*
P0DOY2 (IGLC2)	P06681 (C2)	0.6099	89	0.4601	0.7259	<.0001*
P23141 (CES1)	P05451 (REG1A)	0.1540	89	-0.0560	0.3510	0.1495
P23141 (CES1)	P06681 (C2)	0.3786	89	0.1849	0.5439	0.0003*
P23141 (CES1)	P0DOY2 (IGLC2)	0.1111	89	-0.0994	0.3121	0.2999
P32942 (ICAM3)	P05451 (REG1A)	0.3571	89	0.1608	0.5262	0.0006*
P32942 (ICAM3)	P06681 (C2)	0.6750	89	0.5430	0.7744	<.0001*
P32942 (ICAM3)	P0DOY2 (IGLC2)	0.3739	89	0.1796	0.5401	0.0003*
P32942 (ICAM3)	P23141 (CES1)	0.7123	89	0.5918	0.8016	<.0001*
P42785 (PRCP)	P05451 (REG1A)	0.2746	89	0.0704	0.4568	0.0092*
P42785 (PRCP)	P06681 (C2)	0.6224	89	0.4759	0.7354	<.0001*
P42785 (PRCP)	P0DOY2 (IGLC2)	0.3296	89	0.1303	0.5033	0.0016*
P42785 (PRCP)	P23141 (CES1)	0.7906	89	0.6971	0.8577	<.0001*
P42785 (PRCP)	P32942 (ICAM3)	0.8267	89	0.7470	0.8829	<.0001*

Table S5. Parameters obtained with ROC analysis with each of the six identified proteins to distinguish between the three groups of patients.

NC vs DN						
**Parameter**	**Protein**					
	**P0DOY2 (IGLC2)**	**P05451 (REG1A)**	**P32942 (ICAM3)**	**P06681 (C2)**	**P42785 (PRCP)**	**P23141 (CES1)**
AUC	0.6391	0.7000	0.7494	0.7747	0.7655	0.7897
P†	0.0311	0.0055	0.0008	0.0007	0.0005	<0.0001
Cut-off	9.79	10.32	7.04	10.23	3.65	7.40
Sensitivity, %	66	69	86	83	72	86
Specificity, %	63	67	60	63	83	60
PPV, %	63	67	68	69	81	68
NPV, %	66	69	82	79	76	82
FDR, %	37	33	32	31	19	32

DC vs DN						
**Parameter**	**Protein**					
	**P0DOY2 (IGLC2)**	**P05451 (REG1A)**	**P32942 (ICAM3)**	**P06681 (C2)**	**P42785 (PRCP)**	**P23141 (CES1)**
AUC	0.7022	0.6644	0.6789	0.5378	0.6700	0.6967
P†	0.0289	0.0340	0.0109	0.3370	0.0127	0.0036
Cut-off	10.28	10.66	7.14	10.73	3.65	6.95
Sensitivity, %	80	60	50	27	83	70
Specificity, %	67	70	83	97	53	63
PPV, %	71	67	75	89	64	66
NPV, %	77	64	63	57	76	68
FDR, %	29	33	25	11	36	34

DC vs NC						
**Parameter**	**Protein**					
	**P0DOY2 (IGLC2)**	**P05451 (REG1A)**	**P32942 (ICAM3)**	**P06681 (C2)**	**P42785 (PRCP)**	**P23141 (CES1)**
AUC	0.7885	0.8310	0.6012	0.7908	0.6460	0.6437
P†	0.0002	<0.0001	0.2890	0.0022	0.1101	0.0764
Cut-off	10.24	10.43	6.94	10.17	3.41	6.03
Sensitivity, %	86	72	79	72	52	48
Specificity, %	70	87	43	83	80	83
PPV, %	74	84	58	81	71	74
NPV, %	84	76	68	76	63	63
FDR, %	26	16	43	19	29	26

† Model significance, chi-square test.

Table S6. Performance of ROC analysis on prediction of eGFR impairment (eGFR<60 ml/min) considering all the subjects together. Results are presented for the model including all six proteins, and separately for the three proteins resulted as main contributors in the overall logistic regression.

**Parameter**	**All 6 proteins**	**P0DOY2 (IGLC2)**	**P42785 (PRCP)**	**P05451 (REG1A)**
AUC	0.89	0.81	0.58	0.72
P†	0.0003	0.0006	0.1697	0.0074
Sensitivity, %	92	83	100	92
Specificity, %	83	82	24	50
PPV, %	48	43	18	23
NPV, %	98	97	100	97
FDR, %	52	57	82	77
Probability threshold	0.17	0.17	0.10	0.10
Cut-off for NPX value	--	10.39	4.36	10.51

† Model significance, chi-square test.

Table S7. Correlation of protein P42785 (PRCP) with BMI and glycemia (FPG) in the three groups of subjects. *P* value highlighted in red color and marked with an asterisk indicates significant correlation.

Group	Variable	by Variable	Correlation	Count	Lower 95%	Upper 95%	Signif Prob
DC	P42785	FPG	0.0488	30	-0.3171	0.4020	0.7981
DC	P42785	BMI	-0.0751	30	-0.4239	0.2931	0.6934
DN	P42785	FPG	-0.0560	30	-0.4081	0.3105	0.7687
DN	P42785	BMI	-0.3957	30	-0.6617	-0.0414	0.0304*
NC	P42785	FPG	-0.1300	29	-0.4739	0.2484	0.5016
NC	P42785	BMI	0.2825	29	-0.0937	0.5881	0.1376

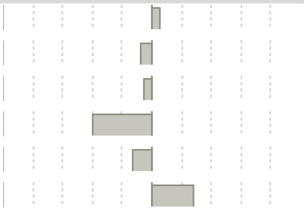

Supplement: Supplementary file 1 [file proteomes-12-00029-s001.zip › proteomes-3158837-supplementary.pdf]
